# Structure Design and Heat Transfer Performance Analysis of a Novel Composite Phase Change Active Cooling Channel Wall for Hypersonic Aircraft

**DOI:** 10.3390/mi15050623

**Published:** 2024-05-06

**Authors:** Weichen Li, Jieliang Zhao, Xiangbing Wu, Lulu Liang, Wenzhong Wang, Shaoze Yan

**Affiliations:** 1 School of Mechanical Engineering, Beijing Institute of Technology, Beijing 100081, China; 3120210421@bit.edu.cn (W.L.); 18801165131@163.com (X.W.); lianglulu12345@163.com (L.L.); wangwzhong@bit.edu.cn (W.W.); 2 Division of Intelligent and Biomechanical Systems, State Key Laboratory of Tribology in Advanced Equipment, Department of Mechanical Engineering, Tsinghua University, Beijing 100084, China; yansz@mail.tsinghua.edu.cn

**Keywords:** active cooling thermal protection, honeycomb structure, topological optimization, phase change material, heat transfer performance

## Abstract

Efficient and stable heat dissipation structure is crucial for improving the convective heat transfer performance of thermal protection systems (TPSs) for hypersonic aircraft. However, the heat dissipation wall of the current TPS is limited by a single material and structure, inefficiently dissipating the large amount of accumulated heat generated during the high-speed maneuvering flight of hypersonic aircraft. Here, a convection cooling channel structure of TPS is proposed, which is an innovative multi-level structure inspired by the natural honeycomb. An active cooling channel (PCM-HC) is designed by using a variable-density topology optimization method and filled with phase change material (PCM). Numerical simulations are used to investigate the thermal performance of the PCM-HC wall, focusing on the influence of PCM properties, structural geometric parameters, and PCM types on heat transfer characteristics. The results demonstrate that the honeycomb-like convection cooling channel wall, combined with PCM latent heat of phase change, exhibits superior heat dissipation capability. With a heat flux input of 50 kW/m^2^, the maximum temperature on the inner wall of PCM-HC is reduced by 12 K to 20 K. Different PCMs have opposing effects on heat transfer performance due to their distinct thermophysical properties. This work can provide a theoretical basis for the design of high-efficiency cooling channel, improving the heat dissipation performance in the TPS of hypersonic aircraft.

## 1. Introduction

Hypersonic aircraft have gradually become a research hotspot in the field of aerospace due to their high maneuverability [1] and strong penetration capability [2]. However, when the aircraft cruises at speeds exceeding 5 Ma in near space, it will be subjected to severe aerodynamic heating [3] and air viscosity friction [4], resulting in surface temperatures of over one thousand degrees Celsius [5,6]. Although most of the heat can be isolated by the Thermal Protection System (TPS) of the aircraft, the remaining aerodynamic heat transmitted to the inside of the structure will cause internal thermal strain and result in an increase in the temperature of the cabin, which will affect the normal use of the thermally sensitive equipment and change the mechanical properties of the structure’s materials, thus affecting its strength and stiffness [7,8]. Therefore, it is necessary to develop a convective cooling structure that can both bear external load and actively cool internally to better dissipate the heat inside the aircraft’s structure.

The fundamental structure of thermal protection systems comprises an outer high-temperature structural layer and an internal non-load-bearing thermal insulation layer [9,10]. The structural layer, composed of high-temperature-resistant metals or composite materials, is designed to withstand external aerodynamic loads, while the thermal insulation layer, consisting of insulating materials applied as coatings and skins, serves to isolate the substantial heat generated by aerodynamic heating. Given that hypersonic vehicles endure prolonged exposure to high-heat-flux aerodynamic thermal environments, coupled with stringent requirements for flight speed and structural integrity, thermal protection structures often exhibit considerable thickness, thereby unavoidably introducing issues of increased mass that adversely impact flight velocity and escalate fuel consumption [11]. Existing convective cooling channels have been employed in scenarios characterized by high heat flux density, such as in the supersonic combustion ramjet engines of hypersonic vehicles [12], where adjustments to channel geometry and dimensions enhance heat dissipation capabilities. Similarly, hypersonic vehicles’ surfaces confront severe thermal loading environments, prompting the integration of convective cooling channels into TPS to replace bulky thermal insulation materials with high-efficiency heat transfer mechanisms. This approach significantly aids in reducing vehicle mass while preserving structural load-bearing capabilities.

Presently, active TPS are predominantly employed in hypersonic vehicles, leveraging active cooling techniques to remove greater amounts of heat, thereby minimizing the limitations imposed by insufficient material heat resistance and mitigating structural damage to the vehicle [13]. According to different cooling methods, active TPS can be divided into three types: sweat cooling, thin film cooling, and convective cooling [14]. Liu et al. [15] designed a sweating-type TPS, which played a role in reducing the structural temperature to some extent, and the sweating cooling reduced the internal structure temperature of the test model by 50 K. Zhou et al. [16] experimentally verified the influence of low compressibility and the ratio of coolant to heat flux density on the thin film cooling performance of the model. Lee et al. [17] numerically studied the thermal performance of a pin fin heat sink and reduced its thermal resistance by 11% and weight by 30% by optimizing the fin density. Among these active cooling technologies, convective cooling, as a clean and renewable cooling method, has received particular attention and become one of the effective thermal protection methods for hypersonic aircraft in long-term service. In addition to the above features, one of the most attractive features of convective cooling (regenerative cooling) is the use of fuel as a coolant, which not only avoids coolant waste but also achieves efficient and environmentally friendly utilization of residual heat energy [18,19].

However, traditional convective cooling techniques also have certain problems, including low heat dissipation, large coolant consumption, and susceptibility to failure [20,21]. Eckert et al. [22] studied the coolant consumption of three cooling methods, namely impinging cooling, convective cooling, and thin film cooling, under the same mainstream conditions. It was found that convective cooling has the highest coolant consumption among the three cooling methods: under laminar flow conditions, convective cooling is 3 times higher than sweating cooling, and under turbulent flow conditions, it is 1.5 times higher than sweating cooling. This also results in a larger weight of the convective cooling system, and its application is mainly concentrated on heat sinks for miniature devices such as electronic components [23], posing significant technical challenges for TPS applications in spacecraft [24]. Thus far, researchers have conducted some studies on the application of convective cooling technology in the aerospace field, including the use of cold liquid hydrogen circuits for engine cooling in aerospace vehicles and its application in TPS for SSTO hot zones [25]. These strategies can enhance fluid perturbation and heat transfer in convective cooling structures, but they cannot fundamentally solve the aforementioned issues associated with convective cooling technology.

In order to enhance the heat dissipation capability of convective cooling channels and improve the load-bearing performance of the channels themselves, natural honeycomb structures have come into our focus. The honeycomb structure itself exhibits excellent mechanical properties, and moreover, the temperature of the honeycomb can maintain dynamic stability, varying between 25 and 45 °C according to environmental temperatures [26]. However, existing research has mostly focused on the macroscopic structure of honeycombs, with less attention given to the biomimetic utilization value of the microstructure of honeycombs. Scholars have conducted relevant research on the structural characteristics and component properties of honeycombs. Zhang et al. characterized the multilevel structure of natural honeycombs at different scales in detail [27,28,29], and used ESEM, AFM, and other methods to observe and analyze the microstructure and composition of the honeycomb, revealing the micro-morphology of the layered composite material composed of beeswax and bee silk. Zhang also studied the principles of anisotropy and temperature control in honeycomb structures through experiments and numerical simulations, proposing a novel porous material design with a surface composite coating. By mimicking the microstructure of honeycombs, this design breaks through the limitation of only biomimicry from a macroscopic perspective. Duan [30,31] proposed honeycomb equivalent mechanical models and expansion performance models, analyzing the mechanical properties of honeycombs at different temperatures. It was found that bees improve the mechanical performance of honeycombs by cyclically using bee silk, which affects the internal heat transfer capacity of the honeycomb and thereby regulates the temperature of the honeycomb.

The honeycomb structure provides an excellent biomimetic object for the design of convective cooling channels. Recently, many scholars have conducted research on the biomimetic heat exchange structure of honeycombs. Lu [32] used a corrugated wall model to evaluate the heat transfer of microporous aluminum honeycomb under convective cooling in a compact heat exchanger, obtaining the optimal honeycomb cell morphology to achieve maximum overall heat transfer efficiency. Wen [33] studied the performance of sandwich metal honeycomb structures under forced air convection conditions through experiments and numerical methods, testing honeycomb structures made of different materials and with different topological structures. The results showed that the total pressure drop was related to the specific surface area density and the honeycomb cell shape, while the total heat transfer rate was related to the specific surface area density, cell shape, and thermal conductivity of the solid material. Pei et al. [34] proposed a honeycomb-shaped ultra-compact plate heat exchanger and studied its flow and heat transfer characteristics, obtaining the optimal geometric structure of the heat exchanger. Therefore, the honeycomb structure has great potential for application in the field of heat exchange, and it is necessary to break through the limitations of traditional porous materials and design convective cooling structures from the perspective of honeycomb microstructure in order to achieve better heat transfer effects.

With the help of microstructure features, the honeycomb achieves a more reasonable heat distribution through heat transfer, thereby improving the heat exchange capacity between the honeycomb walls and the external environment. In order to better apply the honeycomb’s ability to convective cooling channel wall heat transfer, the phase change latent heat characteristics of phase change materials (PCM) can effectively delay the heat transfer on the wall surface [35], allowing more heat to accumulate on the aerodynamic heated flow side of the channel and avoiding transfer to the far end, thus improving the heat transfer efficiency with the fuel medium. PCM has been widely used in fields such as aerospace and battery thermal management. Kim et al. [36] proposed a new type of spacecraft thermal control hardware combined with PCM and studied the thermal carrying capacity of PCM through simulation. Wang et al. [37] studied the influence of composite PCMs composed of expanded graphite and paraffin with different melting points on battery thermal management performance at different phase change temperatures, and improved the efficiency of PCM through numerical optimization.

In recent years, topology optimization methods have gradually become a new way of designing heat transfer structures [38,39,40]. By satisfying conditions such as objective functions and design variables, topology optimization seeks the optimal material layout in the design domain to achieve a certain performance optimum [41]. Tang et al. [38] proposed a topology optimization model for steady-state nonlinear heat conduction structures under large temperature gradients and found that solid material variability affects topology optimization configurations. Zhuang et al. [42] studied the topology optimization problem of transient heat conduction structures with the integration of temperature gradients at fixed time intervals as the objective function. Xia et al. [43] combined the level set method with bi-directional evolutionary structural optimization to perform heat transfer topology optimization on a flat plate. However, the aforementioned studies commonly focus on optimizing thermal parameters to enhance the thermal characteristics of structures, with little consideration given to the load-bearing properties of thermal structures. In the field of statistics, there is a paucity of research related to topology optimization with the objective of minimizing flexibility.

Topology optimization has also found application in the design of biomimetic honeycomb heat transfer structures. Zhou [44] developed a honeycomb structure with cooling channels, employing topology optimization based on parameters such as thermal shielding and dissipation to study its thermomechanical behavior. Li et al. [45] proposed a heterogeneous honeycomb structure and established a single-ring topology optimization model, successfully reducing its thermal elastic deformation. Huang et al. [46], through the MTO optimization method, adjusted the thermal conductivity and convective area to design a honeycomb structure with high cooling performance. While honeycomb structures hold great potential in the field of heat transfer structures, existing topology optimization designs only mimic the macrostructure of natural honeycombs, namely by using homogeneous pore walls without microstructure to imitate their hexagonal structure. It is necessary to break through the limitations of traditional porous materials and conduct topology optimization design from the perspective of honeycomb microstructure to achieve better heat transfer efficiency.

In order to better utilize the biomimetic value of honeycomb microstructure, this study combines biomimetics with topology optimization methods to biomimetically design honeycomb structures at multiple scales, considering both the macroscopic and microscopic features of natural honeycombs. By integrating PCM with the honeycomb structure and utilizing the latent heat storage characteristics of PCM, a novel composite phase change material-honeycomb channel (PCM-HC) is designed. The thermal performance of the PCM-HC’s wall is investigated, taking into account its heat transfer properties.

The remaining sections of this paper are organized as follows. Section 2 presents the construction of a biomimetic honeycomb convective cooling channel (HC) and outlines the micro-design principles for HC. Section 3 introduces a density-based topology optimization method based on the SIMP interpolation model, which is used for two-dimensional cross-sectional topology optimization of HC. Based on this, a multi-scale structural design method called PCM-HC is proposed. In Section 4, a transient heat transfer analysis model for HC and PCM-HC is established using the finite element method (FE). Section 5 analyzes the influence of PCM presence, geometric parameter variations, and the use of different PCMs on the wall heat transfer performance of PCM-HC. Finally, Section 6 provides a summary of the main findings of this study.

## 2. Biomimetic Honeycomb HC Model

Honeycombs possess distinctive structural characteristics that are highly suitable for biomimetic emulation. For instance, they are designed with minimal material usage while maintaining stability, making them an ideal choice for porous structures. Additionally, the inherent insulation capability of honeycombs is worthy of extensive attention. Honeybees employ an ingenious architectural strategy by incorporating propolis as an additive into beeswax, forming the cell walls of honeycombs. This facilitates the regulation of hive temperature in response to a decrease in external environmental temperature. Furthermore, bees cover the surface of their nests with a detachable covering, which serves as passive insulation to regulate temperature. This mechanism helps prevent heat loss from the interior and protects against excessive external heat disturbance [47]. Therefore, at the macroscopic level, the hexagonal shape of the honeycomb is chosen as its overall configuration to enhance the mechanical strength of the convective cooling channel. Building upon this, at the microscopic level, the structural characteristics of biomimetic honeycomb cell walls, resembling layered composite materials, are utilized, with PCM filling the channel interstices, aiming to improve the heat transfer performance of the channel.

### 2.1. HC Model Construction

**Figure 1:**
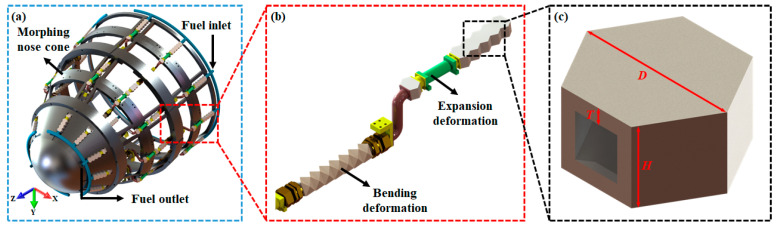
HC model diagram. (**a**) The distribution pattern of HC on the surface of the morphing nose cone. (**b**) The connection method between HC and other functional structures. (**c**) The model structure of HC, with dimensions specified as *D* × *H* × *T* (20 mm × 10 mm × 2 mm).

As shown in Figure 1c, leveraging the macroscopic hexagonal configuration of natural honeycombs, a design model of a honeycomb-inspired convective cooling channel, denoted as HC, was proposed based on the multi-level structure of honeycomb. The HC has a hexagonal shape with dimensions of *D* × *H* × *T* (20 mm × 10 mm × 2 mm) and includes internal cooling channels for fuel flow to achieve convective heat transfer. In the application scenario of hypersonic variant aircraft nose cones, multiple HC units and other channel structures including stretching and bending deformation modules (Figure 1b) are connected to the internal skeleton structure of the aircraft morphing nose cone (Figure 1a) to remove the aerodynamic heat conducted from the skin to the internal structure. This design strategy can effectively improve the heat management performance of the hypersonic aircraft. Subsequent sections primarily focus on the heat transfer performance analysis of the biomimetic honeycomb convective cooling channel. The analysis excludes consideration of the remaining modified duct structures based on variant head cones.

### 2.2. Micro-Design Principles of HC

**Figure 2:**
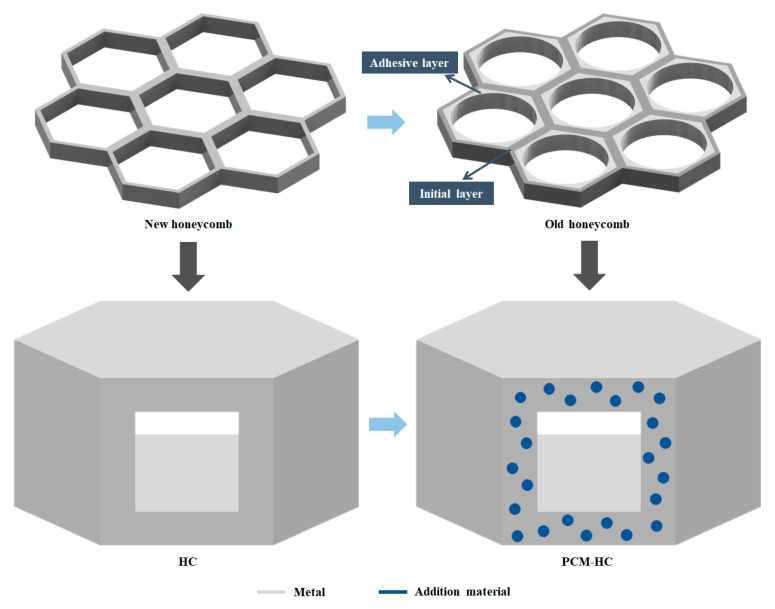
The micro-design method for HC. The HC originates from the new honeycomb, whereas the old honeycomb consists of an initial layer and adhesive layer. Utilizing metal materials as the substrate, and incorporating additional materials, a layered composite structure is designed for PCM-HC, mimicking the layered composite structure of the old honeycomb.

The pore walls of natural honeycombs consist of two parts: the initial layer and the adhesive layer. The initial layer corresponds to the new honeycomb constructed during the initial stages of use, while the adhesive layer gradually forms during the honeycomb’s usage. Consequently, the cell walls of natural honeycombs are considered as fiber composite materials composed of beeswax and bee silk. Taking into account the microstructure and mechanical properties of natural honeycombs, bees enhance the thermal stability of honeycombs by adding bee silk to the surface of new honeycomb walls [28]. Therefore, the design principles for the microstructure of HC walls are illustrated in Figure 2, mimicking the microstructural characteristics of natural honeycombs for HC wall design. Firstly, suitable metal materials are chosen to fabricate HC channels, representing the base material of natural honeycombs, beeswax. Subsequently, utilizing topology optimization methods, parts of the base material are reasonably removed and replaced with other materials (i.e., PCM) to form a micro-layered composite structure similar to honeycomb pore walls.

## 3. Topology Optimization Design of PCM-HC

### 3.1. Topology Optimization Method

A density-based method for topology optimization of continuum structures was employed. The optimization objective was to maximize stiffness while maintaining the optimal HC bearing characteristics by removing some wall material and filling it with PCM to improve the heat transfer capacity of the wall. The density-based method assumes that the material density of each element is different. Material optimization is achieved through independent variation of the relative density of the material. The mathematical model is given as follows [48]:

(1) $$\begin{array}{c} \mathrm{Find}:\rho =\{\rho_{1},\rho_{2},\cdots ,\rho_{n}\}\in R,\\ \mathrm{Minimize}:C=\frac{1}{2}U^{T}KU,\\ \mathrm{Subject}\;\mathrm{to}:KU=F,\\ \rho_{i}\in [0,1],\forall_{i},\\ g=\sum_{i}\rho_{i}-V_{0}\leq 0, \end{array}$$

where *ρ*, *R*, *U*, *K*, *F*, and *V*_0_ are the design variable, the design domain, the displacement matrix, the stiffness matrix, the load matrix, and the target optimized volume, respectively.

In topology optimization, the objective of maximizing stiffness is translated into minimizing the elastic strain energy expressed by the objective function *C* using matrix notation.

The material interpolation models can be classified into two methods: the RAMP method and the SIMP method. These two models are quite similar, but the SIMP method exerts slightly stronger penalization on the element density. The material interpolation model based on the SIMP formulation is given as follows [49]:

(2) $$E_{i}=p(\eta_{i})E_{i_{0}}=\eta_{i}^{p}E_{i_{0}}$$

where $E_{i_{o}}$ is the elastic modulus of the *i*-th element in the fully dense state ($\eta_{i}=1$), $P(\eta_{i})$ denotes the interpolation function, and *p* is the penalty coefficient, which is set to 3.0 [48].

Under the condition of inertial load being overloaded, the classical SIMP model may lead to distortion in low-density elements due to the mismatch between material mass and stiffness models. To address this issue, a polynomial interpolation model can be employed to mitigate the occurrence of element distortion:

(3) $$E_{i}=[(1-\omega )\eta_{i}^{p}+\omega \eta_{i}]E_{i_{0}}$$

where *ω* is the weight coefficient of the linear term. Therefore, the ratio between the penalty coefficient of element mass and stiffness is defined as follows:

(4) $$r_{mK}=\frac{1}{(1-\omega )\eta_{i}^{p-1}+\omega }$$

By incorporating *ω* in the denominator, regardless of the value of $\eta_{i}$ within the range of [0, 1], $r_{mK}$ remains finite in size, resolving the issue of element distortion in the SIMP model.

### 3.2. Topology Optimization Process

**Figure 3:**
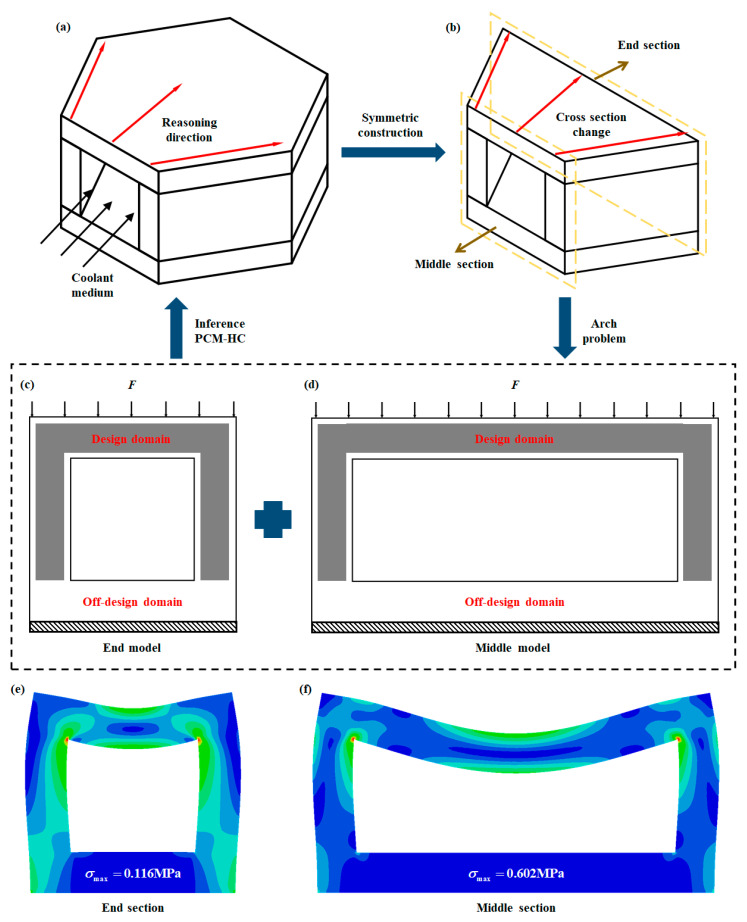
The topology optimization analysis process of HC is transformed from a 3D problem to a 2D problem. (**a**,**b**) HC model simplification process. The HC structure is symmetrical and includes an internal channel for coolant flow, which is simplified into a 2D arch-shaped problem. (**c**,**d**) Topological optimization model. Two cross-sections, one located at the end and another at the middle of the structure, are selected for topology optimization. (**e**,**f**) Stress contour plots for the end section and middle section are presented. The difference of color in different regions of the cross section represents the difference of stress magnitude. The maximum stress values for the end section and middle section are 0.116 MPa and 0.602 MPa, respectively.

The HC channel is a symmetrical structure (Figure 3a) that can be split in half along the symmetry plane, while the 2D cross section gradually lengthens into an arch-like structure along the axial direction. The stress distribution of the 2D cross section is similar, allowing the 3D problem to be simplified to a 2D problem. Therefore, the optimization of the structural topology was carried out on two cross sections of the HC channel, namely at the end and middle sections, and the result was extrapolated to the 3D topology structure (Figure 3b).

Utilizing the topology optimization module (ATOM) in Abaqus for structural topology optimization, a 2D cross-sectional topology optimization model was established. The bottom surfaces of both end sections and the midsection were fully constrained, while the upper surface was subjected to a uniformly distributed load F. Considering the practical application scenario of PCM-HC heat pipes, the model distinguished between the design domain and non-design domain. A boundary layer within 0.2 mm of the outer contour of the wall was frozen (Figure 3c,d). Within ATOM, stress, volume fraction, and weighted strain energy were initially defined as responses to be constrained or targeted. Stress constraints were based on the compressive strength of SUS304 (515 MPa), with an upper limit of 500 MPa. Volume fraction constraints were set as design variables, with volume fraction gradients of 40%, 55%, and 70% (representing the ratio of optimized volume to original volume). The weighted strain energy was selected as the objective function, with the optimization direction set to minimize. Simultaneously, in the geometric constraint module, a minimum size constraint of 0.1 was defined to prevent the generation of overly thin structures, thereby achieving more uniformly distributed material distribution in the optimized results.

The static stress contour maps of the end and middle sections of the HC channel in Figure 3e,f show that the stress concentration is mainly located around the middle region of the upper wall surface and at the diagonal intersection between the upper wall and side walls. These regions should be preserved as the main load-bearing parts in the design of the PCM-HC channel. As the bottom surface of the end and middle sections serves as the farthest point of thermal conduction and exhibits low stress, this area will become the main removal region in the topology optimization process and is therefore designated as the non-design domain. Additionally, the maximum stress on the compressed side wall of the middle section is approximately 5.2 times greater than that of the end section. Therefore, the topology optimization result of the middle section was selected as the overall design reference for the PCM-HC channel.

**Figure 4:**
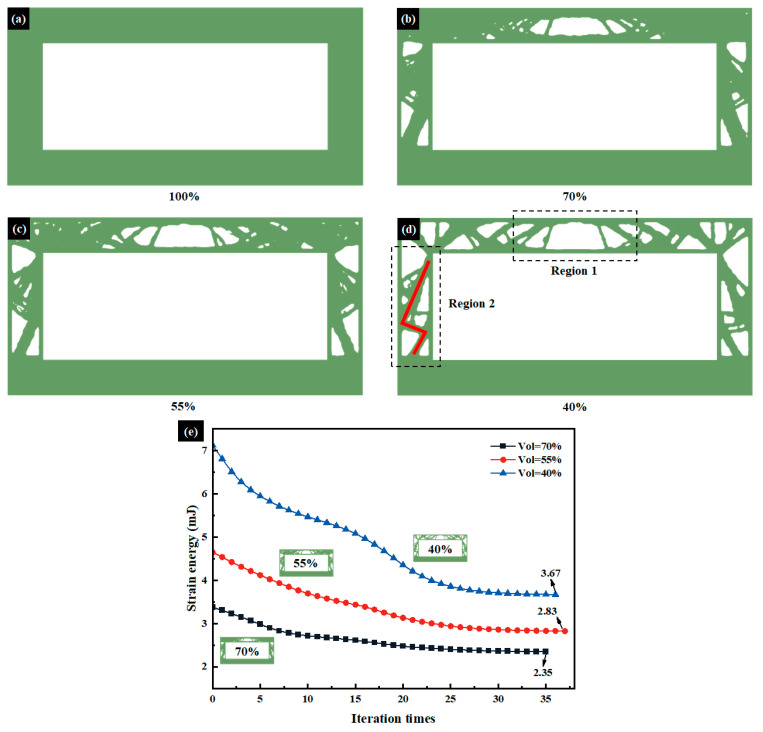
Topology optimization results of the middle section. (**a**) The initial structure diagram of the middle section. (**b**–**d**) Topology optimization results under volume fraction constraints of 70%, 55%, and 40%. Under a volume fraction constraint of 40%, region 1 exhibits a trapezoidal structure, while region 2 forms a “Z”-shaped pattern. (**e**) Variation of strain energy with iterations at different volume fractions.

In Figure 4a–d, topology optimization is performed with volume fraction gradients set as 100%, 70%, 55%, and 40%, indicating that the optimization mainly focuses on areas of low stress in the structural design region. Therefore, topology optimization primarily optimizes the lowest-performing units in the overall structural performance area by removing inefficient units in order to improve the overall performance and mechanical properties of the structure. As the volume fraction changes, the outline of the main load-bearing structure becomes clearer: for the upper wall surface, the material reduction is mainly concentrated in the middle Section 1, with a trapezoidal shape, and symmetrically distributed along the cross-sectional axis. For the side wall surface, with a volume fraction of 40%, a “Z”-shaped support structure is clearly observed in area 2, indicating that this design method can enhance the lateral load-bearing capacity of the overall structure.

In order to compare the mechanical strength of HC sections at different volume fractions, Figure 4e illustrates the variation of strain energy with topology optimization iterations for volume fractions of 70%, 55%, and 40%. Strain energy serves as a measure of structural stiffness, where minimizing strain energy is equivalent to maximizing overall stiffness. As observed in the figure, with an increase in iterations, the optimization effectiveness of HC sections’ stiffness improves progressively. At the conclusion of optimization, the strain energy of HC sections with volume fractions of 40% and 55% exceeds that of HC sections with a volume fraction of 70% by 56.2% and 20.4%, respectively. Thus, as the volume fraction decreases, the structural stiffness of HC sections diminishes. Consequently, when the volume fraction is 70%, the mechanical strength is optimal.

### 3.3. Determination of PCM-HC Model

**Figure 5:**
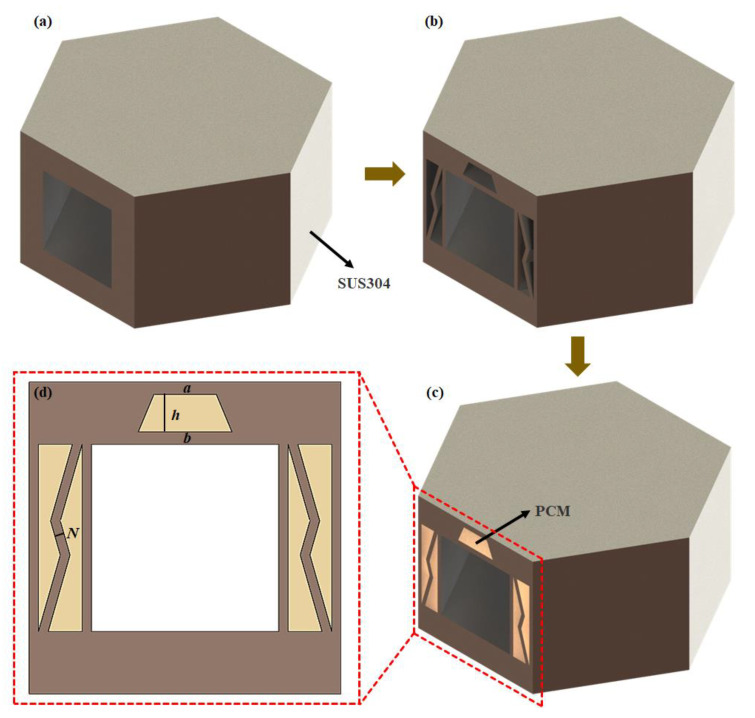
The process of establishing the PCM-HC model. The HC structure (**a**) is modified by removing a portion of SUS304 material (**b**) and filling it with PCM to obtain a PCM-HC structure (**c**). (**d**) The trapezoidal PCM area height is *h*, the upper bottom length is *a*, the lower bottom length is *b*, and the web thickness is *N*.

Based on the topology optimization analysis results of the middle section, a gradual removal of the substrate material SUS304 (Figure 5b) along the cross-sectional direction of HC (Figure 5a) is performed. Subsequently, the cavity is filled with PCM to obtain the PCM-HC model (Figure 5c). In Figure 5d, the belly plate thickness (*N*) on the side wall varies between 0.15 mm, 0.30 mm, 0.45 mm, and 0.60 mm, while the PCM region on the upper wall has a trapezoidal shape. The characteristic parameters of the trapezoidal PCM region are determined based on the trapezoid area formula S=(a+b)h/2. The trapezoidal PCM cross section characteristic vector is defined as follows:

(5) $$W_{i}=(\alpha_{i},\beta_{i},h_{i})=(\frac{a_{i}}{h_{i}},\frac{b_{i}}{h_{i}},h_{i}),\;\;i=1,2,\ldots ,n$$

where $a_{i}$ is the length of the upper base of the trapezoid, $b_{i}$ is the length of the lower base of the trapezoid, and $h_{i}$ is the height of the trapezoid.

At this point, the area of the trapezoidal PCM region can be expressed as follows:

(6) $$S=\frac{1}{2}(\alpha_{i}+\beta_{i})h_{i}^{2}$$

Keeping the area of the trapezoidal PCM region *S* = 6 mm^2^ constant, let $W_{1}=(\frac{10}{3},5,\frac{6}{5})$, $W_{2}=(\frac{5}{3},\frac{20}{3},\frac{6}{5})$, $W_{3}=(4,8,1)$, $W_{4}=(2,10,1)$.

**Figure 6:**
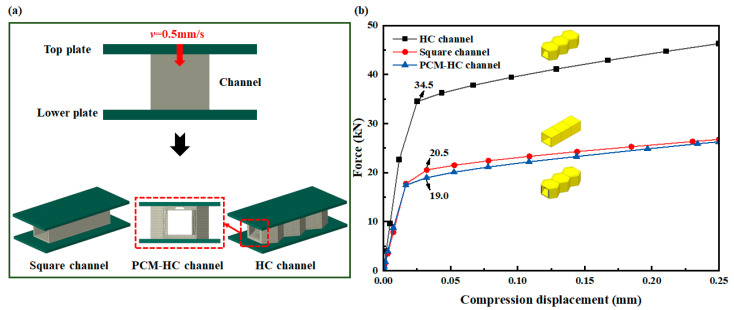
The finite element models and mechanical property analysis of the channel. (**a**) The finite element models of the HC channel, PCM-HC channel, and square channel maintain the same load-bearing area and cavity volume. (**b**) The variation of load capacity with displacement for the HC channel, PCM-HC channel, and square channel under axial compression between rigid plates is investigated.

When hypersonic vehicles cruise in near-space, they will experience severe aerodynamic loads, imposing significant structural demands on the internal convective cooling channels. The HC and PCM-HC model, inspired by the high mechanical performance of honeycomb structures, are compared with traditional square channels through a simulation of their mechanical properties to verify the structural load-bearing capacity. The square channels maintained the same load-bearing area and internal volume as the HC and PCM-HC models (Figure 6a).

Mechanical analysis was conducted using the commercial finite element software Abaqus 2021. As depicted in Figure 6a, the finite element model of the channel was placed between upper and lower rigid plates. The lower end of the rigid plate was fixed in all degrees of freedom, while the upper rigid plate compressed downward at a constant speed of 0.5 mm/s. The contact between the upper and lower rigid plates and the channel model was defined as general contact, with tangential penalty contact, a friction coefficient of 0.2, normal hard contact, and allowed separation after contact. The material of channels was defined as SUS304, with material parameters listed in Table 1. HC and square channels are discretized using eight-node linear hexahedral elements (C3D8R), while the PCM-HC channel employs ten-node quadratic tetrahedral elements (C3D10) for discretization, culminating in the completion of the computational solution.

The force-displacement curves under axial compression for the channel models depicted in Figure 6b reveal three distinct stages of deformation, namely elastic, yielding, and plastic deformation, for HC, PCM-HC, and square channels. With increasing axial displacement, the mechanical characteristic curves of the models exhibit a gradual upward trend. However, HC demonstrates significantly higher axial load-bearing capacity compared to square and PCM-HC channels, with peak test forces at yield strengths of 34.5 kN, 20.5 kN, and 19.0 kN, respectively. HC exhibits a 68% increase in load-bearing capacity over the square channel under identical bearing area and heat transfer area conditions. Even after removing some material to accommodate PCM, HC maintains comparable load-bearing performance to the traditional square channel.

## 4. Research on PCM-HC Performance

### 4.1. Physical Model Description

Due to the uniform heat flux conditions imposed on the upper surface of the convective cooling channel, both the HC and PCM-HC channels, as symmetric three-dimensional structures, can be simplified into a two-dimensional cross-sectional problem, where heat conduction along the length of the channel can offset each other. Under the same heat flux input conditions, if the temperature of the walls surrounding the channel is lower, the heat transfer burden on the cooling medium inside the channel is reduced. Establishing a two-dimensional cross-sectional model of the channel, the thermal distribution on the walls is studied in the absence of coolant flow, aiming to minimize the heat transfer burden on the convective cooling channel as much as possible.

**Figure 7:**
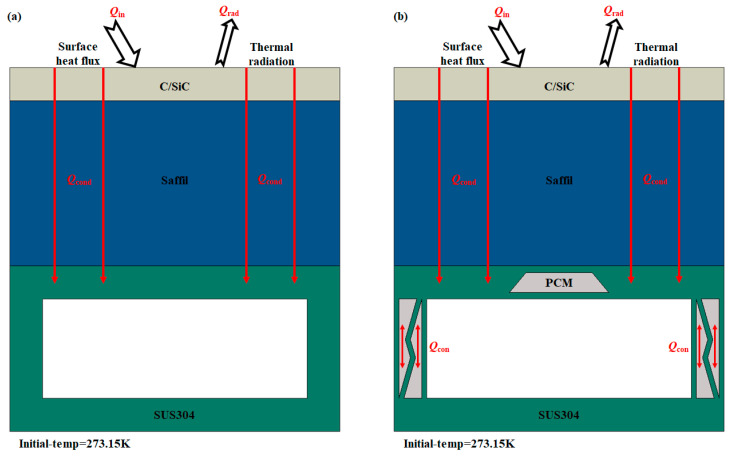
Transient heat transfer analysis models for HC and PCM-HC. In HC (**a**) and PCM-HC (**b**), *Q*_in_ represents the input heat flux load, *Q*_rad_ denotes the surface thermal radiation of the system, *Q*_cond_ signifies the internal heat conduction within the system, and *Q*_con_ represents the convective heat transfer within the PCM. The arrows represent the direction of heat transfer. The initial temperature of the system is 273.15 K.

To better simulate the practical application scenario of convective cooling channels, a conventional multi-layer TPS structure is introduced above the HC and PCM-HC structures. A thermal load is applied to the outer surface of the system to simulate the aerothermal load experienced by hypersonic aircraft during flight. The multi-layer TPS consists of an outer high-temperature structural layer and an inner non-load-bearing insulation layer, as shown in Figure 7. The upper layer, depicted in earthy yellow, is made of C/SiC composite high-temperature material to withstand the external thermal load *Q*_in_, with a thickness of 2 mm. The middle layer, shown in dark blue, utilizes Saffil alumina fiber insulation material to isolate the significant heat generated by aerothermal effects, with a thickness of 10 mm. The lower layer, represented in deep green, serves as the convective cooling channel with a base material of SUS304. The gray area added in Figure 7b represents the filled PCM. The thermal and physical properties of C/SiC and Saffil refer to relevant literature [50,51], with some parameters varying with temperature.

For the HC model, the heat transfer modes considered include thermal radiation *Q*_rad_ on the top surface and internal heat conduction *Q*_cond_ within the structure. For the PCM-HC model, the heat transfer modes include thermal radiation *Q*_rad_ on the top surface, internal heat conduction, and convective heat transfer *Q*_con_ which arises only within the PCM due to the phase change process. In addition, the contact thermal resistance between different material interfaces is ignored, and each contact surface is treated as an ideal heat transfer surface. Assuming the liquid phase of the PCM behaves as an incompressible fluid, the volumetric force effects resulting from PCM phase change are neglected [52,53].

### 4.2. Heat Transfer Mathematical Model

The following equations represent the governing equations for HC and PCM-HC systems, including continuity, energy, and momentum equations, which are described as follows:

(a) Continuity equation [54]:

(7) $$\frac{\partial \rho }{\partial t}+\nabla (\rho \vec{v})=0$$

where *ρ* is density and $\vec{v}$ is is velocity vector.

(b) Energy equation [55,56,57]:

(8) $$\frac{\partial (\rho E)}{\partial t}+\nabla (\rho \vec{v}E)=\nabla (k\nabla T)+S$$

where *E* is the total enthalpy, *k* is the thermal conductivity coefficient, *T* is temperature, and *S* is the energy source term.

Total enthalpy *E* can also be represented as follows:

(9) $$E=E_{0}+\Delta E$$

where $E_{0}$ is sensible enthalpy and ΔE is latent enthalpy.

When the PCM is in a solid state, ΔE is 0. When the PCM is in a liquid state, ΔE is *L*, where *L* is the latent heat of the PCM. When the PCM is in a semi-solid or mushy state, ΔE takes on values between 0 and 1. The liquid fraction function *δ*, which is temperature-dependent, is defined as follows [58]:

(10) $$\delta (T)=\frac{\Delta E}{L}=\left\{ \begin{array}{l} 0,T<T_{s}\\ \frac{\left(T-T_{s}\right)}{\left(T_{l}-T_{s}\right)},T_{s}\leq T\leq \\ 1,T>T_{l} \end{array}\right.T_{l}$$

where $T_{s}$ denotes the lower limit of the phase transition temperature range and $T_{l}$ denotes the upper limit of the phase transition temperature range.

In Equation (8), *S* is equal to the sum of the internal heat source and radiative flux, i.e.,

(11) $$S=\nabla (\rho \vec{v}\Delta E)+\nabla \vec{Q_{rad}}$$

When there is no internal heat source, the energy source term *S* is equal to the radiative flux. $\vec{Q_{rad}}$ represents the presence of outward thermal radiation on the upper surface of the system, which is defined by the following boundary condition equation [56]:

(12) $$\vec{Q_{rad}}=k\frac{\partial T}{\partial y}=Q_{in}-\sigma \varepsilon (T_{w}^{4}-T_{e}^{4})$$

where *σ* is the Stefan–Boltzmann constant, *σ* = 5.67 × 10^−8^ W/(m^2^·K^4^). $Q_{in}$ is input heat flux density, *ε* is radiance, and $T_{w}$ is wall temperature. $T_{e}$ is ambient temperature, according to the International Standard Atmosphere (ISA), and values are determined for different flight altitudes.

(c) Momentum equation [54]:

(13) $$\frac{\partial \vec{v}}{\partial t}+\nabla (\vec{v}\vec{v})=-\frac{1}{p}\nabla P+\frac{1}{p}\nabla^{2}(\mu \nabla \vec{v})+\vec{F}$$

where *μ* is dynamic viscosity, *P* is pressure, and $\vec{F}$ is momentum source term.

When PCM undergoes a liquid phase change, the expression of dynamic viscosity *μ* is as follows [59,60]:

(14) $$\mu =0.001e^{\left(A+\frac{B}{T}\right)}$$

where *A* is −4.25 and *B* is 1790 K.

Furthermore, in the analysis of transient heat transfer problems involving PCM, the enthalpy-porosity method is employed to calculate the phase change process [61]. In Equation (13), the momentum source term $\vec{F}$ is defined as follows:

(15) $$\vec{F}=\frac{c_{m}{\left(1-\delta \right)}^{2}}{\left(\delta^{3}+\eta \right)}\vec{v},$$

where $c_{m}$ is a mushy zone constant that is between 10^5^ and 10^7^ [62,63]. The introduction of *η* is to prevent division by zero. The function *δ*, mentioned earlier, represents the temperature-dependent liquid fraction.

### 4.3. Numerical Simulation Program

Transient heat transfer analysis of HC and PCM-HC was conducted using the Abaqus finite element software. Structured grids were employed, and a series of 2D models were established to analyze the heat transfer capability of the convective cooling channel when using PCM. The grid type used was DC2D4, with local refinement near the PCM region. The total number of grids was 5059. By solving the two-dimensional equilibrium equations at each node, the temperature distribution at various nodes was calculated, and a temperature contour plot of the model was generated.

As for the boundary conditions, a uniform heat flux load *Q*_in_ was applied to the top surface of the system, with a loading time of 600 s. Additional time calculations were performed to obtain the maximum temperature on the bottom wall of the channel. The outer surface is subjected to aerodynamic loads, while the two sides and bottom are situated within the aircraft with minimal gap areas where convection effects can be neglected. Therefore, both sides and the bottom of the system are set as adiabatic boundary conditions [56]. Furthermore, there was outward thermal radiation on the top surface with an emissivity (*ε*) value of 0.85. Additionally, the initial temperature condition for the entire system was set as 273.15 K.

## 5. Result and Discussion

Firstly, a comparison was made between the heat transfer characteristics of the convective cooling channel with and without the use of PCM filling. Then, in the presence of PCM, the combined effects of two parameters, namely the trapezoidal PCM cross section characteristic vector (**W**) and the belly plate thickness (*N*), were evaluated. In this study, five input heat flux densities were considered (30 kW/m^2^, 35 kW/m^2^, 40 kW/m^2^, 45 kW/m^2^, and 50 kW/m^2^). Lastly, three types of PCM, namely Erythritol, Urea, and Paraffin composite phase change material (CPCM), were selected to investigate their respective impacts on heat transfer.

### 5.1. Effect of PCM on Heat Transfer Performance

In this section, the heat transfer performance of convective cooling channels with and without PCM filling was compared for five different input heat flux densities (30 kW/m^2^, 35 kW/m^2^, 40 kW/m^2^, 45 kW/m^2^, and 50 kW/m^2^). Two channel system models were established to compare the effect of PCM. The only difference between them was that the HC channel consisted entirely of SUS304 while the PCM-HC channel was composed of SUS304 and Erythritol. The input heat flux was uniformly applied to the surface of the system, and heat was transferred within the TPS and channel walls primarily through conduction and radiation, as detailed in Section 4.1. It should be noted that in this study, the trapezoidal PCM cross section characteristic vector was $\left(\frac{10}{3},5,\frac{6}{5}\right)$, and the belly plate thickness was 0.30 mm.

**Figure 8:**
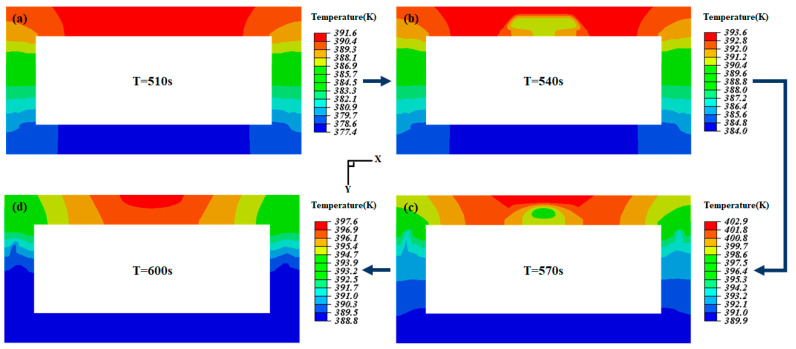
The wall temperature distribution of PCM-HC varies with time under a heat flux load of 50 kW/m^2^. (**a**–**d**) are 510 s, 540 s, 570 s, and 600 s, respectively. The arrows represent the order of time change. $W=(\frac{10}{3},5,\frac{6}{5})$, *N* = 0.30 mn.

Figure 8 illustrates the wall temperature distribution at different time intervals in the PCM-HC channel under a heat flux load of 50 kW/m^2^. In general, the heat flow is applied from the upper wall surface, resulting in a gradual decrease in wall temperature from the proximal end of the heat flow input to the distal end. Under the same heat transfer distance, the rate of heat transfer is dependent on the temperature difference between the object and its thermal conductivity, which can be explained by Fourier’s law (q=kΔT/L, where *q* is the transferred heat flux, *k* is the material’s thermal conductivity, ΔT is the temperature difference, and *L* is the distance between the heat exchange surfaces). At 510 s, the heat is uniformly diffused from the upper wall surface to both side wall surfaces, with a temperature difference of 14.2 K (Figure 8a). Subsequently, as the PCM reaches its phase change temperature, a significant amount of heat is absorbed during the phase change process. The heat transfer in the trapezoidal PCM region differs from the surrounding area, resulting in lower temperatures and a decreased rate of temperature rise, leading to almost constant temperatures (Figure 8b). At the same time, the PCM on the side walls also undergoes phase change and absorbs heat, resulting in a noticeable delay in temperature rise and a decrease in temperature gradient within the side walls (Figure 8c). At 540 s and 570 s, the temperature difference between the highest and lowest temperatures is reduced to 12.5 K and 13 K, respectively, as the phase change latent heat gradually decreases and the heat dissipation capacity reaches its limit. At 600 s, the PCM completes the phase change process, and the rate of temperature rise gradually recovers, with a reduced temperature difference of 8.8 K between the highest and lowest temperatures (Figure 8d).

**Figure 9:**
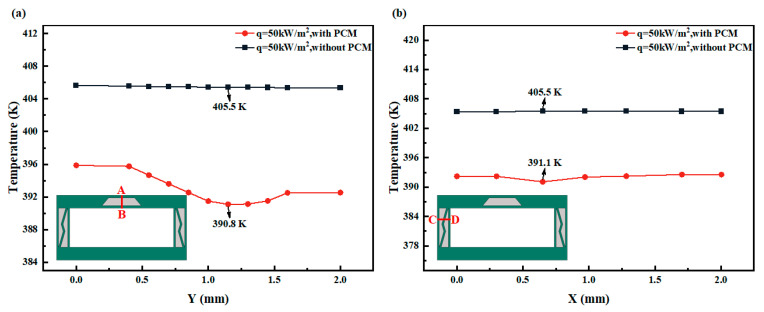
The temperature distribution along a specified path at a specific moment with and without PCM was examined. (**a**) The red line AB indicates the temperature distribution along the vertical trapezoidal PCM at 570 s on the upper wall surface. (**b**) The red line CD indicates the temperature distribution along the horizontal PCM at 590 s on the side wall surface.

As shown in Figure 9, the temperature variations along the AB path at 570 s and the CD path at 590 s under a heat flux load of 50 kW/m^2^ are selected to demonstrate the thermal dissipation capability of PCM in the PCM-HC system. In the upper wall surface (Figure 9a), in the non-PCM region, the heat conduction rate is uniform and decreases slowly. However, in the PCM region, the temperature is significantly lower than in the non-PCM region, and the rate of temperature change accelerates. The temperature exhibits a decreasing trend followed by an increasing trend, with the lowest temperature dropping by nearly 5 K compared to the highest temperature in the non-PCM region. Additionally, compared to the HC channel without PCM, the PCM-HC system exhibits an average temperature reduction of 10 K to 15 K. Similarly, the side wall surface (Figure 9b) displays the same pattern of temperature variation. Due to the smaller area covered by PCM, the temperature drop in the PCM region is less pronounced. Nevertheless, it still demonstrates superior thermal dissipation capability compared to HC alone, with an average temperature reduction of 13 K. Thus, in the PCM-HC system, heat conduction near the PCM is slowed down, resulting in lower temperatures compared to the same location in the HC system and impeding heat transfer.

**Figure 10:**
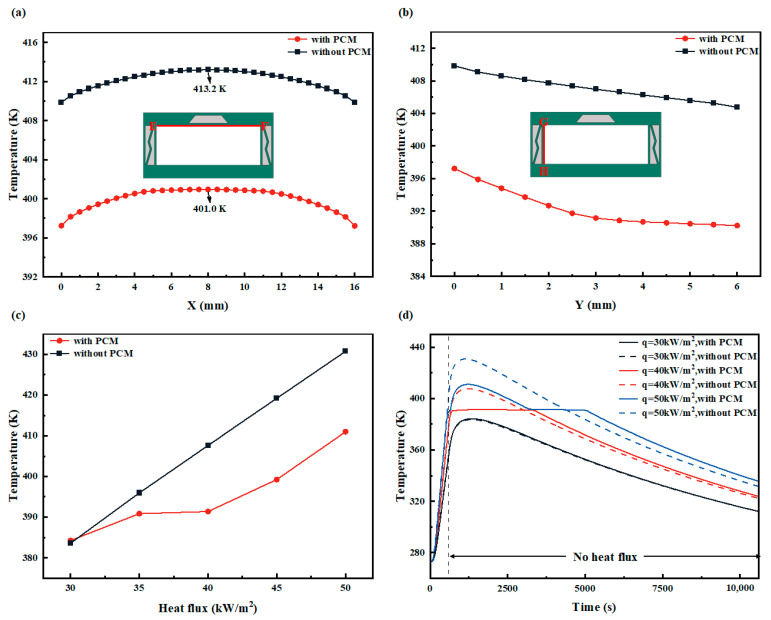
The effect of PCM on the heat transfer performance of HC and PCM-HC. (**a**) The temperature distribution of the upper wall (EF red line). (**b**) The temperature distribution of the side wall (GH red line). (**c**) With the increase in heat flux input (*Q*_in_ = 30, 35, 40, 45, and 50 kW/m^2^), the maximum temperature of the lower wall changes. (**d**) *Q*_in_ = 30, 40, and 50 kW/m^2^; the temperature variation of the lower wall during the entire loading and unloading process after 600 s was recorded.

Figure 10a,b illustrate the temperature variations along the EF path (upper wall surface) and CD path (side wall surface), respectively, at the end of the heat flux loading for HC and PCM-HC under a 50 kW/m^2^ heat flux input (at 600 s). These paths represent the inner wall surfaces involved in convective heat transfer with the fuel coolant, and a lower temperature distribution is desirable to reduce the burden of heat transfer with the fuel coolant. On the upper wall surface, both HC and PCM-HC exhibit a similar temperature trend, gradually increasing from the sides towards the center, with a temperature difference of approximately 4 K. This is because as the heat flux loading time increases, the phase change process of PCM is completed, leading to a loss in its ability to block heat transfer. The overall temperature of PCM-HC on the upper wall surface is about 12 K lower than that of HC. On the side wall surface, the temperature decrease in PCM-HC is faster than HC in the first half, while in the second half, the temperature decrease in PCM-HC is slower than HC. This is because PCM in the side wall surface is undergoing a phase change, absorbing a significant amount of heat due to latent heat, which attracts the heat flux from the upper wall surface and hinders heat conduction to the lower wall surface. The overall temperature of PCM-HC on the side wall surface is approximately 15 K lower than that of HC.

Figure 10c shows the variation of the maximum temperature on the lower wall surface with and without PCM in the convective cooling channel. As shown in the figure, as the heat flux input increases, the temperature difference between HC and PCM-HC undergoes a change from almost zero to gradually increasing and then maintaining a certain level. This is because when the heat flux input is small, within the 600 s loading time, PCM has not reached its phase change temperature and therefore cannot play a role in heat dissipation. With the increase in heat flux input, after the end of the 600 s heat flux loading, the remaining heat supply enables PCM to slowly reach its phase change temperature, thus exerting a certain heat dissipation effect. Finally, when the heat flux input continues to increase to a level that can quickly meet the latent heat of PCM’s phase change, PCM fully exerts its heat dissipation effect. When the heat flux input is 45 kW/m^2^, the heat dissipation effect is close to saturation, and the maximum temperature on the lower wall surface with PCM is about 20 K lower than that without PCM. This trend indicates that different scenarios of convective cooling channels correspond to different input heat fluxes and heating durations. Thus, it is necessary to select PCM with a suitable phase change temperature according to the specific requirements to fully utilize its heat dissipation capability.

Figure 10d illustrates the variation trend of the lower wall temperature during the entire loading and unloading process of PCM-HC and HC at different heat flux inputs for 600 s. As the heat flux input increases, both with and without the heat flow input, PCM exhibits good thermal conductivity. When the heat flux input is relatively small, there is no significant difference in temperature between the cases with and without PCM throughout the process, since the PCM has not reached its phase change temperature. When the heat flux input increases to 50 kW/m^2^, the highest temperature of the lower wall decreases from 402.7 K to 389.1 K with PCM under heat flow input, and from 430.8 K to 411.0 K without PCM under heat flow input. This reveals that the use of PCM to enhance the heat conduction of the wall in the convective cooling channel is suitable only for high heat flux inputs. Meanwhile, in the section without heat flow input, the temperature begins to drop after reaching its maximum value. As the temperature approaches the phase change temperature of the PCM, the PCM begins to store energy in the form of latent heat, and the temperature remains almost unchanged for a duration of 2000 s, delaying the temperature drop. After the PCM fully solidifies, further heat conduction causes the temperature to continue to decrease, resulting in a higher temperature of the channel with PCM than that without PCM, with a maximum temperature difference of 7.5 K. This implies that the solidification phase change of the PCM during temperature drop is crucial for maintaining the internal temperature stability of the spacecraft when it loses its heat source, consistent with the insulating function of natural honeycomb structures.

Therefore, based on the above analysis, the phase change latent heat of PCM effectively reduces the surface temperature when heat is loaded, improves the heat distribution, promotes heat conduction, and reduces the burden of convective heat transfer between the fuel coolant and channel wall in the convective cooling channel. At the same time, the phase change latent heat of PCM also plays a role in thermal insulation for the spacecraft as the system temperature drops. Therefore, the combination of PCM is an effective approach for designing convective cooling channels with excellent heat conduction capabilities.

### 5.2. Effect of Geometric Parameter on Heat Transfer Performance

#### 5.2.1. Effect of Trapezoidal PCM Cross Section Characteristic Vector (W)

In this section, the effect of changing the characteristic vector of the trapezoidal PCM ($W_{1}=(\frac{10}{3},5,\frac{6}{5})$, $W_{2}=(\frac{5}{3},\frac{20}{3},\frac{6}{5})$, $W_{3}=(4,8,1)$, $W_{4}=(2,10,1)$) in PCM-HC was assessed while keeping the PCM area and position unchanged. Other control parameters were kept constant, *Q*_in_ = 50 kW/m^2^.

**Figure 11:**
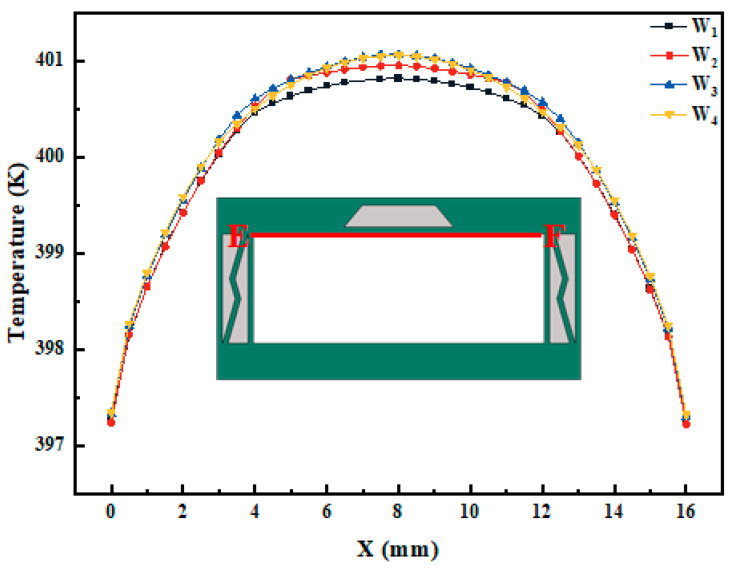
The effect of trapezoidal PCM cross section characteristic vector change ($W_{1}=(\frac{10}{3},5,\frac{6}{5})$, $W_{2}=(\frac{5}{3},\frac{20}{3},\frac{6}{5})$, $W_{3}=(4,8,1)$, $W_{4}=(2,10,1)$) on the temperature distribution on the upper wall of PCM-HC under a heat flux input of 50 kW/m^2^.

The graph in Figure 11 illustrates the temperature variation along the EF path (upper wall) at the end of the thermal load (600 s) for different trapezoidal PCM cross section characteristic vectors under a heat flux input of 50 kW/m^2^. It can be observed that the temperature distribution is symmetrical, and changes in characteristic vectors have minimal impact on the temperature at the edges of the wall but affect the thermal distribution at the middle position of the wall. As the height h of the trapezoid increases, the temperature at the middle position decreases. Additionally, increasing the difference in length between the upper and lower bases of the trapezoid also leads to a decrease in the middle position temperature. When **W** = (2,10,1), the temperature difference between the highest and lowest temperatures at the middle position is 0.3 K. Although the overall dimensions of the model influence the magnitude of temperature differences, it also indicates that the trapezoidal PCM characteristic vector provides optimal heat dissipation. We also studied the influence of trapezoidal PCM cross section characteristic vectors on the thermal distribution and heat dissipation on the side and bottom walls. However, since the temperature hardly changes, we will not provide detailed explanations here.

#### 5.2.2. Effect of Belly Plate Thickness (*N*)

In this section, the effect of belly plate thickness (*N* = 0.15 mm, 0.30 mm, 0.45 mm, 0.60 mm) on heat transfer in PCM-HC will be investigated under the fixed trapezoidal PCM cross section characteristic vector of **W** = (2,10,1), while keeping the other control parameters constant, *Q*_in_ = 50 kW/m^2^.

**Figure 12:**
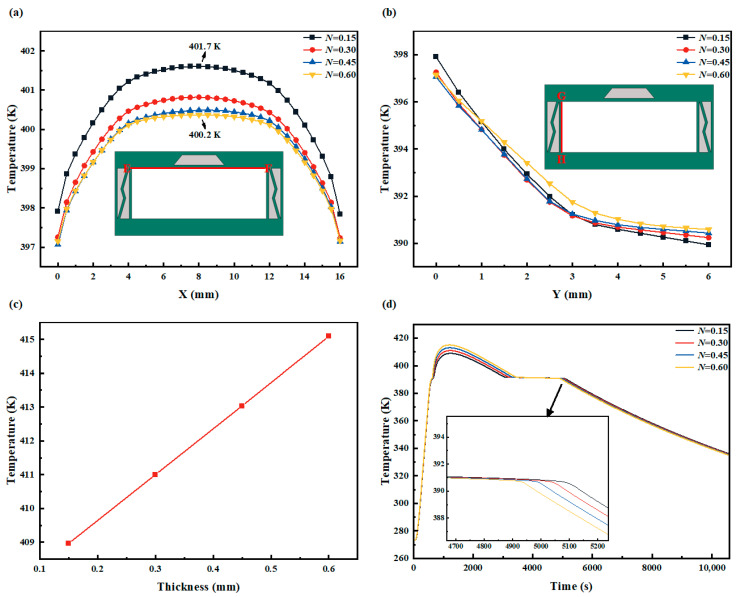
The effect of belly plate thickness changes (*N* = 0.15, 0.30, 0.45, and 0.60 mm) on the heat transfer performance of PCM-HC (*Q*_in_ = 50 kW/m^2^, **W** = (2,10,1)). (**a**) The temperature distribution variation on the upper wall (EF red line). (**b**) The temperature distribution variation on the side wall (GH red line). (**c**) The variation of the maximum temperature on the lower wall with increasing belly plate thickness *N*. (**d**) The temperature variation on the lower wall during the entire loading and unloading process after 600 s.

Figure 12a illustrates that, under the same heat flux input, the temperature on the upper wall decreases with an increase in the baffle thickness. However, this decrease is not uniform; rather, it diminishes less as the thickness increases. This phenomenon arises because the increase in baffle thickness leads to an increase in the volume of the base SUS304 and a decrease in the PCM volume. SUS304 exhibits better thermal conductivity, facilitating faster heat transfer from the upper wall downwards. Simultaneously, the reduction in PCM volume reduces the heat absorption during PCM phase change, weakening its heat dissipation effect and thereby allowing heat to reach the side and lower walls more rapidly, resulting in a decrease in temperature on the upper wall. However, there exists a balance point, due to the limited heat conduction speed in the upper wall region, where the increase in baffle thickness causing accelerated heat conduction reaches equilibrium, hence reducing the decrease in temperature on the upper wall. The maximum temperature difference between baffle thicknesses of 0.60 mm and 0.15 mm is approximately 1.5 K.

Additionally, in Figure 12b, under the same heat flux input, except for *N* = 0.60 mm, the temperature on the side wall decreases in the upper half section and increases in the lower half section with an increase in the baffle thickness. The maximum temperature difference between baffle thicknesses of 0.60 mm and 0.15 mm exceeds 0.5 K. The variation inside wall temperature distribution is analogous to that of the upper wall, wherein the combined effects of SUS304 and PCM result in non-uniform changes in heat distribution with changing baffle thickness. Increasing baffle thickness accelerates heat transfer towards the channel’s far end, yet weakens PCM’s attraction to heat flux on the upper wall and its hindrance to heat transfer on the lower wall simultaneously.

Figure 12c illustrates the trend of the maximum temperature on the lower wall of the PCM-HC channel as the baffle thickness increases, showing a nearly linear relationship. The temperature difference between *N* = 0.60 mm and *N* = 0.15 mm is 6 K. This is attributed to the constant thickness of the left and right support pillars on the side wall, resulting in an increase in PCM and a decrease in SUS304 as the baffle thickness decreases. Compared to SUS304, a larger area of PCM enhances the heat dissipation capacity of the channel. PCM possesses latent heat of phase change and a larger specific heat capacity, meaning that when the area of the PCM-HC channel remains constant, PCM can absorb more heat at much lower temperatures. Simultaneously, PCM’s latent heat of phase change absorbs more heat, resulting in a lower temperature on the lower wall.

Figure 12d depicts the variation trend of the lower wall temperature throughout the entire process of removing the heat load after 600 s of thermal loading under different baffle thicknesses in the PCM-HC channel. It can be observed that as the baffle thickness decreases, the heat dissipation effect of PCM, both with and without heat flux input, improves. Comparing *N* = 0.15 mm to *N* = 0.60 mm, with heat flux input, the maximum temperature on the lower wall decreases from 390.4 K to 389.5 K, and without heat flux input, it decreases from 415.1 K to 409.0 K. This indicates that adjusting the baffle thickness to enhance heat conduction on the wall of the convective cooling channel yields favorable results.

In summary, reducing the thickness of the baffle significantly decreases the temperature of the lower wall while causing a slight increase in temperature in certain regions of the upper and side walls. However, this increase is minimal compared to the temperature drop on the lower wall and can be considered negligible. Therefore, overall, reducing the baffle thickness is conducive to enhancing the heat dissipation capability of the PCM-HC channel. However, it is imperative to consider the mechanical strength of PCM-HC; decreasing the baffle thickness will inevitably diminish the channel’s load-bearing performance. Consequently, the actual baffle thickness should be selected based on the specific load-bearing requirements of the channel. Hence, taking into account both heat transfer and load-bearing performance, a compromise is made in the test design, with a relatively optimal baffle thickness of 0.30 mm chosen.

### 5.3. Effect of PCM Species on Heat Transfer Performance

In this section, the effect of using different PCMs on PCM-HC heat transfer will be studied under the optimal geometric combination with a fixed trapezoidal PCM cross section characteristic vector of **W** = (2,10,1) and a fixed belly plate thickness of *N* = 0.30 mm, while keeping other control parameters constant and *Q*_in_ = 50 kW/m^2^. To achieve better thermal management performance, PCMs with low thermal conductivity, high latent heat of phase change, and high specific heat capacity are preferred, provided that the PCM phase change temperature is lower than the actual temperature inside the system. Erythritol, Urea, and Paraffin CPCM were selected as the three PCM options, and their thermophysical properties [64,65,66] are listed in Table 2.

**Figure 13:**
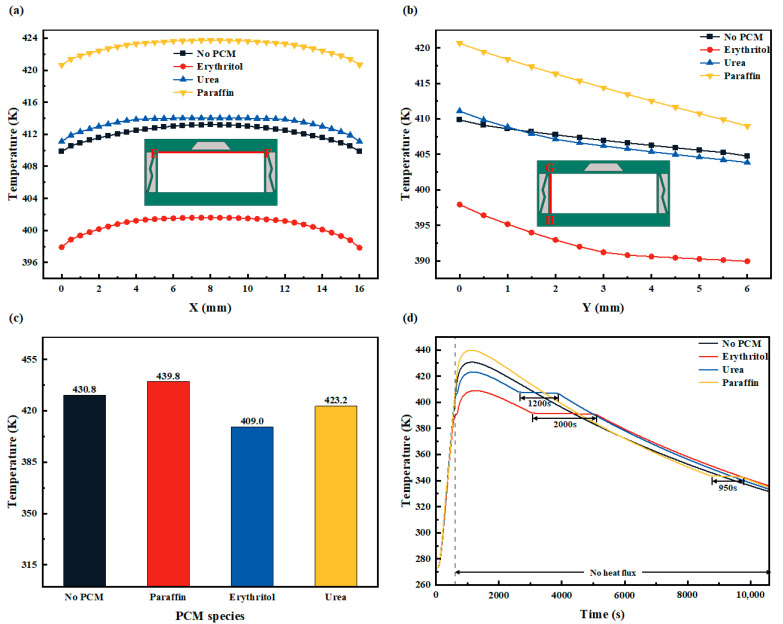
The effect of different PCMs (Erythritol, Urea, and Paraffin CPCM) on the heat transfer properties of PCM-HC (*Q*_in_ = 50 kW/m^2^, **W** *=* (2,10,1), *N* = 0.30 mm). (**a**) The variation of temperature distribution on the upper wall (EF red line). (**b**) The variation of temperature distribution on the side wall (GH red line). (**c**) Comparison of the highest temperature on the bottom surface for different PCMs. (**d**) Variation trend of the temperature on the lower wall during the entire process of removing the heat load after 600 s of loading with different PCM fillings.

As shown in Figure 13a,b, the filling of different PCMs has produced varying effects on the temperature distribution of the upper and side walls of PCM-HC. Erythritol, with its higher specific heat capacity and phase change heat and lower thermal conductivity compared to the other two PCMs, exhibits excellent thermal conductivity. Compared to the HC without PCM, the average temperature of the upper wall decreases by 12 K, and the average temperature of the side wall decreases by 15 K when using Erythritol. On the other hand, Urea shows poor thermal conductivity due to its higher phase change temperature relative to Erythritol and lower phase change latent heat. Within the limited heat flux loading time, the upper wall completes the phase change latent heat process early and continues to release sensible heat, while some regions of the PCM in the side wall do not reach the phase change temperature, resulting in the incomplete utilization of PCM’s thermal conductivity ability. As a result, the average temperature of the upper wall increases by 1.2 K, and the temperature of the side wall decreases by 0.5 K. The addition of Paraffin CPCM has the opposite effect on the system, leading to an increase in the temperatures of both the upper and side walls. The average temperature of the upper wall rises by nearly 11 K, and the average temperature of the side wall increases by 7.5 K. This is because Paraffin CPCM has a lower phase change temperature. At *Q*_in_ = 50 kW/m^2^ and within a loading time of 600 s, PCM-HC completes the phase change and releases sensible heat much earlier. Additionally, the highest temperature on the lower wall of the PCM-HC channel in Figure 13c reflects the different effects of the three PCMs analyzed above. Compared to the HC without PCM, Erythritol, Urea, and Paraffin CPCM exhibit temperature changes of a decrease by 21.8 K, a decrease by 7.6 K, and an increase by 9 K, respectively.

Figure 13d shows the variation trend of the temperature on the lower wall of PCM-HC during the entire process of removing the heat load after 600 s of loading with different PCM fillings. A comparison reveals that Erythritol exhibits the best thermal conductivity of the PCM-HC structure. During the heat flux loading stage, the highest temperature on the bottom surface decreases by 13 K. In the absence of heat flux loading, the highest temperature on the bottom surface decreases by 20 K. Meanwhile, during the phase change process without heat flux input, the temperature remains almost unchanged for a certain period of time, which is approximately 2000 s for Erythritol, 1200 s for Urea, and 950 s for Paraffin CPCM. This indicates a delayed temperature decrease and demonstrates that Erythritol provides the best insulation effect among the three PCMs.

Therefore, the analysis of heat transfer performance for different PCMs demonstrates that not all PCMs are beneficial for improving the thermal conductivity of PCM-HC. In summary, when using PCM-HC, it is necessary to select a PCM with suitable phase change temperature and latent heat according to the structural parameters and material properties of TPS, as well as the size and duration of the input heat flux load.

## 6. Conclusions

In this paper, a novel convective cooling channel filled with PCM is proposed. The introduction of PCM aims to improve the heat dissipation capability of the channel, that is, to reduce the wall temperature of the channel. Firstly, a model of a honeycomb-like convective cooling channel (HC) was proposed. Then, two-dimensional topology optimization of HC was conducted using the variable density method based on the SIMP interpolation model, and the PCM-filled PCM-HC structure was constructed. The mechanical load-bearing capacities of HC, PCM-HC, and square channels are then compared. Finally, numerical analysis of heat transfer was carried out from three aspects, i.e., with and without PCM, variation of PCM geometric parameters, and use of different types of PCMs, to determine the optimal geometric combination of the designed convective cooling channel (trapezoidal PCM cross section characteristic vector of (2,10,1), and a thickness of 0.15 mm for the belly plate). The main conclusions are as follows: (1) The convective cooling channel with PCM filling exhibits excellent thermal conductivity. The melting phase change of PCM changes the heat distribution, effectively reducing the wall temperature of the channel and reducing the burden of convective heat transfer between fuel and channel wall. Under the maximum heat flux load input of the tested design, the highest temperature on the inner wall of the channel decreased by 12 K to 20 K. Meanwhile, during the process of PCM solidification latent heat, the temperature remains almost constant when the temperature of the spacecraft system drops, providing insulation for the spacecraft. (2) When the heat flux load remains constant and the area of the trapezoidal PCM cross section is kept unchanged, the temperature distribution on the upper wall of the PCM-HC channel shows variations with changes in the characteristic vector parameters. The optimal thermal conductivity of the channel’s upper wall is achieved when the trapezoidal PCM cross section characteristic vector parameters are set to (2,10,1). However, due to the constraints of the overall size of the channel design, this parameter has a less significant impact on the heat transfer performance of the side wall and lower wall. (3) As the thickness of the belly plate decreases from *N* = 0.60 mm to *N* = 0.15 mm, the thermal conductivity of the convective cooling channel exhibits non-uniform variations. Reducing the thickness of the belly plate can significantly lower the temperature of the lower wall of the channel. However, due to the combined effect of the two materials, SUS304 and PCM, in the channel, there is an uncertain impact on the temperature of the side walls. Nevertheless, this impact results in minimal temperature changes. Considering the load requirements of PCM-HC, a belly plate thickness of 0.30 mm is relatively optimal in the tested design, with the highest temperature on the lower wall decreasing by approximately 1 K. (4) Different types of PCMs have different effects on PCM-HC. Erythritol and Urea possess both thermal conductivity and insulation effects. However, Paraffin CPCM did not lower the wall temperature; instead, it increased it, attributed to the thermal physical properties of the PCM. (5) For the convective cooling channel filled with PCM designed in this study for application in the TPS of hypersonic vehicles, it is advantageous for heat dissipation if the difference between the heights of trapezoidal PCM cross-section vectors and the lengths of their upper and lower bases is designed to be greater. Additionally, a smaller thickness of the belly plate is favorable for heat dissipation, provided it meets the channel’s load-bearing requirements. Furthermore, the selection of PCM with appropriate phase change temperature and latent heat should be based on the specific operating conditions of the channel, such as the magnitude of the input heat flux load and the duration of loading, to ensure the full utilization of PCM’s heat dissipation capabilities.

## Figures and Tables

**Table 1:** Material parameters of SUS304.

Material	*ρ*/g∙cm^−3^	*E*/Gpa	*σ*/Mpa	*v*
SUS304	7.93	204	800	0.285

**Table 2:** PCM thermophysical properties.

Property	Paraffin CPCM	Erythritol	Urea
Density (kg/m^3^)	780	1450	1320
Specific heat capacity (J/kg/K)	2350	2610	2100
Thermal conductivity (W/m/K)	1.12	0.73	0.8
Phase transition temperature (K)	343.15	391.15	407.15
Latent heat (kJ/kg)	189.6	340	250

## Data Availability

The research data for this article can be obtained from the corresponding author “Jieliang Zhao” on reasonable request.
